# Variations in Cycling Distances by Trip Purpose and Socio-Demographic Attributes: Implications for Spatial Scales to Assess Environmental Correlates of Cycling

**DOI:** 10.3390/ijerph21121648

**Published:** 2024-12-10

**Authors:** Firas Mohamed, Manoj Chandrabose, Abdur Rahim Mohammad Forkan, Neville Owen, Takemi Sugiyama

**Affiliations:** 1 Centre for Urban Transitions, Swinburne University of Technology, Melbourne 3122, Australia; mchandrabose@swin.edu.au (M.C.); nowen@swin.edu.au (N.O.); tsugiyama@swin.edu.au (T.S.); 2 Department of Mathematical Sciences, South Eastern University of Sri Lanka, Sammanthurai 32200, Sri Lanka; 3 Baker Heart and Diabetes Institute, Melbourne 3004, Australia; 4 School of Science, Computing and Engineering Technology, Swinburne University of Technology, Melbourne 3122, Australia; fforkan@swin.edu.au

**Keywords:** travel survey, physical activity, bicycle use, buffer size

## Abstract

To better understand environmental attributes associated with cycling, it is necessary to identify an area within which such attributes are measured. Various sizes of a “buffer” drawn from home have been used for this purpose. The distances adults cycle to/from their homes may inform the determination of empirically supported buffer sizes. We examined the distribution of cycling distances using Australian travel survey data collected between 2012 and 2020. We used a Random Forest model to identify the relative importance of factors influencing participant’s cycling distance and then reported variations in cycling distances by the most important factors. Of the 73,142 survey participants who were aged between 20 and 74 and reported at least one trip on the survey day, 1676 (67% men) reported 3446 home-based cycling trips, with a median distance of 3.5 km. The most important factor was trip purpose, followed by gender. The median distances were 1.8 km for utilitarian, 5.3 km for commuting and 3.7 km for recreational cycling trips. Men cycled longer distances than women, particularly for commuting and recreational cycling. The significant variation in cycling distance by trip purpose implies the need for having purpose-specific spatial scales in identifying environmental attributes associated with cycling more accurately.

## 1. Introduction

Cycling can be a physical activity of moderate-to-vigorous intensity [1] and is known to confer health benefits, including reduced risk of chronic diseases and better mental wellbeing [2,3,4,5,6]. Cycling for transportation can also mitigate traffic congestion, as it requires less space for riding and parking compared to cars [7]. When more people cycle instead of driving, there is reduced air and noise pollution [8,9,10]. Cities that are cycling friendly have less fuel consumption for transportation, providing environmental and economic benefits [11].

Cycling is an accessible mode of transportation for most people with known health benefits, yet its prevalence is very low in many countries. A comparison of 17 countries found that bicycles were used for less than 12% of trips, except in the Netherlands, where nearly one-fourth of trips were made by cycling [12]. Among the 35 major cities investigated in this international study, the cycling mode share was below 4% in more than half of them [12]. There is a need to increase the prevalence of cycling for transport in many cities across the world. 

Evidence from multiple countries shows that certain attributes of the built environment can be associated with cycling behaviours [13,14,15]. Understanding environmental attributes related to cycling is important to inform environmental strategies to produce bicycle-friendly neighbourhoods. An increasing number of studies have examined this issue, and a literature review synthesising the current evidence has been published [16]. In this review, cycling was classified into three types based on trip purposes, namely for transport (travel from one place to another in general), for commuting (travel specifically between home and workplace or school) and for recreation (cycling for leisure or exercise). They found that cycling for transport was associated with street connectivity and availability of local destinations and that cycling for commuting was associated with the presence of cycling paths. However, no strong correlates were identified for recreational cycling. The distinct pattern of findings identified in this review suggests the need for purpose-specific studies in identifying environmental attributes relevant to cycling.

An important issue in examining built environmental correlates of cycling is how to define an area within which environmental attributes are measured. This is relevant as the way such an area is delineated could influence research findings (i.e., whether and to what extent a certain attribute is associated with cycling) [17]. Since cycling normally starts from home, where people keep their bicycle, drawing a “buffer” area around home is one way to define such an area, in which environmental attributes are measured and their associations with cycling behaviours are examined. A key consideration here is the buffer size, i.e., how far the buffer should extend from home. Previous studies investigating built environmental attributes related to cycling have used various buffer sizes, with limited empirical support for the selection of these sizes. For instance, Porter et al. [18] used 1.5 km and 3 km buffers to identify environmental correlates of cycling for transport and for recreation. They chose these distances as they corresponded to 5 min and 10 min cycling distances, but they did not provide justification for these cycling durations. Nielsen et al. [19] used a 1.5 km buffer, using a similar reasoning related to cycling duration. Beenackers et al. [20] and Ma et al. [21] used a 1.6 km buffer, which is often used to define a neighbourhood [22], without explaining why this buffer size was chosen. A few studies employed buffer sizes that were conventionally used in studies examining environmental correlates of walking, such as 500 m, 800 m and 1 km [13,23,24]. However, since bicycles are often used to reach destinations that are too far to walk, environmental characteristics of areas beyond walking distances need to be considered to accurately capture environmental attributes related to cycling. 

To identify appropriate buffer sizes for cycling, evidence on how far people cycle from home can be informative. Such a distance-referenced approach has been employed in informing buffer sizes for studies on the built environment correlates of walking [25,26]. There is limited research on the distances people cycled from home, but the findings are not directly applicable in determining relevant buffer sizes for cycling. For instance, a recent study investigating factors associated with cycling distances in the Netherlands, Denmark and Germany [27] found that the cycling distances varied by gender, age and trip purpose. However, they reported mean cycling distances, which may not be informative for determining a buffer size. They also reported distances for eight specific purposes, which were not comparable to the three major cycling purposes (transport, commuting and recreation) that have been shown to have distinct environmental correlates [16]. Factors associated with cycling distance for commuting were examined in Norway, but the distances that participants cycled were not reported [28]. No research appears to have examined cycling distances in the context of Australia. A better understanding of the distribution of cycling distances from home for distinctive purposes is needed to inform the selection of appropriate spatial scales in identifying environmental attributes associated with cycling.

This study sought to identify how far people cycle from home and its variation using travel survey data collected from two Australian states (Queensland and Victoria). There were two objectives. First, we examined the relative importance of trip purpose and demographic characteristics in explaining adults’ cycling distances. Second, we identified the distribution of cycling distances and its variations by the most important factors identified in the first step. We did not consider environmental attributes, which can affect cycling distances, in this study, because the aim is to provide empirical support in determining buffer sizes within which potential environmental correlates of cycling can be assessed.

## 2. Materials and Methods

### 2.1. Data Source

Data for this study were obtained from two travel surveys in Australia: the Victorian Integrated Survey of Travel & Activity (VISTA) and the Queensland Household Travel Survey (QHTS). Both are ongoing surveys administered by the Department of Transport of the State Government in Victoria and the Department of Transport and Main Roads in Queensland. Data were collected between 2012 and 2020 (before the COVID-19 pandemic). VISTA data were collected from the Melbourne metropolitan area and surrounding regional cities (Geelong, Ballarat, Bendigo, Shepparton and Traralgon). QHTS data were collected from Brisbane; Gold Coast; Sunshine Coast; and regional cities of Cairns, Rockhampton and Toowoomba. Details of these surveys, such as the population of target areas and response rates, are provided in Table S1.

Both surveys used multi-stage random sampling to recruit participants. Mesh blocks, the smallest geographic area in the Australian Statistical Geography Standard [29], were randomly selected before the random selection of households in each selected mesh block. All members of the selected households were invited to participate in the survey. They were asked to complete a self-administered questionnaire about their demographic characteristics. They also reported details of their trips conducted on the designated survey day, including origin, destination, start time, end time, mode and purpose, using a 24 h travel diary. VISTA and QHTS collected data using comparable instruments, which allowed us to combine the databases. Multiple measures to ensure validity of the data were incorporated at the stages of data collection and cleaning, including logic checks built into the questionnaires. Further details of the survey procedures are explained in their respective reports [30,31]. The surveys were conducted in accordance with the ethical guidelines of the government statutes and regulations. The datasets are publicly accessible through the open data portals of their respective states [32,33].

### 2.2. Study Participants

VISTA collected data from 78,978 participants in 30,803 households, while QHTS obtained data from 51,535 participants in 21,204 households. Of the total participants (*n* = 130,513), 26,445 did not travel on the survey day and 952 had missing data for travel behaviours. Of the remaining participants (*n* = 103,116), we focused on those between 20 and 74 years old (*n* = 73,142). Those aged 75+ were excluded, as they may experience potential age-related functional difficulties [34]. The final sample of the study consisted of 1676 participants who reported at least one home-based cycling trip, which was either originated from or terminated at participants’ homes. There were 103 participants who cycled after they left home, presumably using bicycle-sharing schemes. We did not include them in the analyses, since this study sought to identify how far people cycled from home.

### 2.3. Outcome Variable

The outcome measure for this study was the distance of home-based cycling trips. This was estimated using the shortest road-network distance between home and the destination reported in the travel diary [30,35]. If a cycling trip involved multiple destinations, we only considered the distance of trips from home and to home.

### 2.4. Explanatory Variables

The variables used to explain the variability in the cycling distance were trip purpose (see below for details), gender, age group (younger adults: 20–39 years old; middle-aged adults: 40–59 years old; and older adults: 60–74 years old), employment status (working or not working), and geographic region (metropolitan or regional area) and state (Victoria or Queensland) where participant resided. These demographic variables were included as they are conventionally used as individual-level correlates of cycling [27,28]. The geographic variables were added as the level of cycling could be different between these regions. It should be noted that built environment attributes, such as the presence of bike paths, were not considered as explanatory variables, as the objective of the study is to help determine the area within which such attributes need to be examined. Participants reported the purpose of each trip in the travel diary. We categorised trips into utilitarian, commute and recreation purposes, following the classification of cycling used in the review conducted by [16]. We used “utilitarian” instead of “transport” in our category of cycling purpose to differentiate cycling trips to get to local destinations (shops and services) from cycling to work/school. Further details of this classification (specific purposes reported) are provided in Table 1. 

### 2.5. Data Analyses

To provide a comprehensive description of the distribution of cycling distance, we reported the mean, standard deviation (SD), median, 20th percentile (P_20_) and 80th percentile (P_80_) values. Since the distribution of cycling distance was positively skewed, we considered the median value as the typical cycling distance (i.e., covered by half of the trips). The distance corresponding to the point at which 80% of the trips fell below the value (the 80th percentile) was considered as the upper threshold of distance beyond which would be too far for most people to cycle. The 80th percentile was used in previous studies that sought to identify the distance that is walkable [36,37] and bikeable [36] from travel survey data. On the other hand, the 20th percentile of the distance was considered as the lower threshold, below which would be too close for cycling (i.e., walking distance). Since most cycling trips occur within the 80th percentile distance from home, it is possible to argue that this distance can be used to define a buffer for investigating environmental attributes associated with cycling. However, it is unknown whether other distances may be more suitable as a buffer size. Therefore, we also calculated the 75th and 85th percentile of cycling distances.

To examine whether cycling distances varied by the explanatory variables, we performed nonparametric statistical tests, the Wilcoxon signed-rank test and the Kruskal–Wallis test [38]. The former is used to assess the statistical significance of the difference between the medians of two groups, and the latter is used for comparing three groups. These statistical tests were conducted using R Programming Language [39].

A Random Forest model was employed to identify the relative importance of the explanatory variables on cycling distance. Random Forest is a decision tree-based machine learning algorithm that combines the outcomes of multiple decision tree to a single result. It is a widely used, robust method for estimating the relative importance of each explanatory variable in relation to the outcome variable [40,41]. It has been shown that Random Forest models have higher prediction accuracy [42,43] and thus perform better in estimating variable importance than a single decision tree-based model [44]. This approach has recently been used in studies on travel behaviours to assess the relative importance of explanatory variables [45,46,47]. The Random Forest algorithm involves the creation of an ensemble of decision trees, each of which is built on an independent sample of cases chosen from the dataset with replacement, i.e., bootstrapped samples [48]. At each split of a tree in the Random Forest model, only a random subset of explanatory variables is considered as split candidates. This facilitates each explanatory variable, even the weakly correlated ones, with more chance to influence the outcome variable compared to a single decision tree [44]. The decision trees are constructed by recursively partitioning the dataset based on the explanatory variable that minimised the variability of cycling distance [49]. Residual sum of squares is used to measure the reduction in variability at each step of partitioning. We used an actual impurity reduction importance measure to estimate the relative importance of each explanatory variable [50]. The Supplementary Materials provide further details about how the Random Forest was built. We performed the Random Forest modelling in R using the ‘ranger’ package [51], mostly with its default parameter settings unless we specified otherwise here.

## 3. Results

Table 2 shows the characteristics of the 1676 participants who reported at least one home-based cycling trip on the survey day. Two thirds were men, with some 80% being younger or middle-aged. Eighty percent were employed, and almost ninety percent of them resided in metropolitan areas. The proportion of participants who cycled to/from home was 2.3% of the participants who travelled on the survey day. This proportion differed considerably between men (3.2%) and women (1.5%). Older adults (1.9%) had a lower proportion of cyclists in comparison to younger and middle-aged adults.

Table 3 shows the characteristics of the home-based cycling trips. The total number of home-based cycling trips was 3446 (2.1 trips/day on average by each cyclist), and their median distance was 3.5 km (P_20_ =1.3 km, P_80_ = 8.2 km). Cycling trips were almost equally divided into utilitarian (33%), commuting (35%) and recreational (31%) purposes. A significant difference in the cycling distance by trip purpose was found. The median cycling distances were longest for commuting (5.3 km), followed by for recreational (3.7 km) and shortest for utilitarian purposes (1.8 km). Figure S1 shows the distribution of cycling distance for each trip purpose. The median cycling distances were also found to differ to some extent according to the demographic factors examined. The cycling distances were shorter for women, older adults, those who were not working and those who resided in in regional areas or in Queensland. Table S2 show the alternative upper thresholds (the 75th and 85th percentile distances) for each cycling purpose and demographic attribute. It shows that these thresholds produced some variation in the distances. For all the cycling trips, P_75_ was 7.0 km, while P_85_ was 9.7 km.

**Figure 1:**
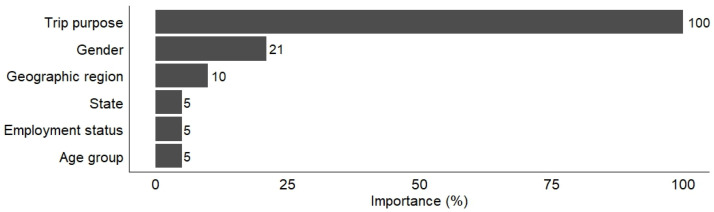
Relative importance scores of the explanatory variables on home-based cycling distance.

Figure 1 shows the relative importance scores of the explanatory variables for cycling distance, obtained from the Random Forest model. The trip purpose was identified as the most important factor explaining the variation in cycling distance and was assigned the score of 100% for comparison purposes. Gender was the second most important determinant (with a relative importance score of 21%), followed by geographic region (10%), state (5%), employment status (5%) and age (5%).

Table 4 shows cycling distances cross-classified by the two most important determinants identified by the Random Forest model: trip purpose and gender. Men’s cycling distance was significantly longer than women’s in all trip purposes, particularly for commuting and recreational trips. For utilitarian trips, the difference between the median cycling distances of men and women (2.0 km for men and 1.6 km for women) was smaller compared to the other trip purposes. 

## 4. Discussion

Using travel survey data from two Australian states (Queensland and Victoria), we examined the distributions of cycling distances travelled by adults to/from their home. Overall, we identified more than 1600 participants who reported such home-based cycling trips. However, they accounted for only 2% of the travel survey participants who made at least one trip on the survey day, indicating the very low prevalence of cycling in Australia. An analysis of these cycling trips found significant differences in cycling distances by trip purpose and, to a lesser extent, by gender. Other socio-demographic and geographic attributes were found to be less important in accounting for the variation in home-based cycling distances.

### 4.1. Comparisons with the Existing Literature

Our findings regarding the key determinants of cycling distance are generally consistent with previous studies conducted in Europe [27,28]. Both studies found trip purpose to be significantly related to cycling distances. Commuting trips were found to be longer than trips for any other purposes both in the study conducted in the Netherlands, Denmark, and Germany [27] and in our study. Consistent with our findings, Schneider et al. [27] also reported the shortest distances for utilitarian cycling trips. Gender was also found to be significant in these studies: men were found to cycle longer distances than women. 

However, our findings differed from those of Schneider et al. [27] in terms of the cycling distances identified. The overall median (2 km) and mean (3 km) distances reported in Schneider et al. [27] were shorter than the respective distances that we found in Australia (median = 3.5 km, mean = 5.3 km). A possible explanation for the differences could be different demographic makeup of cyclists between these studies. Table 2 shows that a higher percent of cycling trips is reported by men in Australia (67%) compared to Europe (45%). Similarly, more middle-aged adults cycled in Australia (42%) than in Europe (36%). This could suggest that cycling in Australia may be more likely to be carried out by people who are physically stronger and can cycle longer. Such differences in cycling distance can mean that the buffer sizes within which environmental attributes are measured may not be generalisable to different contexts in which the characteristics of cyclists are different. Smaller buffer sizes may be applicable to European countries, where cycling is a more common mode of transport undertaken by a broader range of people.

### 4.2. Variations in Cycling Distance by Trip Purpose

Our analysis using a Random Forest model found that the most important factor in explaining the variance in home-based cycling distances was trip purpose. Our findings suggest the need for applying different buffer sizes in examining environmental correlates of cycling for different purposes. This is in contrast to the existing studies, where environmental correlates of cycling were examined without differentiation by cycling purposes [19,21] and using the same buffer sizes for transport and recreational cycling [18,20].

Our findings also suggest that future studies investigating environmental correlates of cycling may need to use larger buffer sizes. The largest buffer size employed in the previous studies was 3 km in Porter et al. [18]. However, we found that the 80th percentile of cycling distance ranged from 4 km for utilitarian cycling to 10 km for commuting cycling. These are the distances within which most cycling trips occur, which means that environmental attributes within these distances from home are likely to be relevant to cycling. However, it is unknown if the 80th percentile distance is the most appropriate buffer size. Future research can examine to what extent environmental attributes measured within different thresholds (e.g., the 75th and 85th percentile distances shown in Table S2) are associated with cycling for specific purposes.

### 4.3. Variations in Cycling Distance by Gender

Gender was found to be the second most important determinant of cycling distances. It was found that women tended not only to use bicycle less but also to cycle shorter distances compared to men. The distances reported for utilitarian trips did not vary substantially by gender, but greater gender differences were found in commute and recreational cycling trips. These findings suggest that gender differences may have to be considered in investigating environmental correlates of commuting and recreational cycling. Given the pressing need to promote cycling for women who are currently less likely to cycle in Australia [12,52], buffers based on the distances women can cycle could be justified. Such buffer sizes may help to identify environmental characteristics that are relevant specifically to women’s cycling and help to reduce gender disparities in cycling.

### 4.4. Strengths and Limitations

We used large-scale travel survey data collected from diverse geographic settings, including metropolitan and regional areas from two states in Australia. The statistics on cycling distance could vary significantly with cycling prevalence because the higher the prevalence, the more diverse the cyclists. Therefore, the findings of our study may be generalisable to other regions with low prevalence of bicycle use but may not be directly applicable to regions with high-cycling prevalence. Further empirical studies from diverse localities could help to better understand cycling distances and their variability. The outcome measure used was not the actual cycling distance, but an estimate based on the shortest road-network distance between origins and destinations. Consequently, this study may have underestimated cycling distances, as some participants might have used different routes (not the shortest) to reach their destinations. Furthermore, cycling trip data used in this study were collected using a 24 h travel diary. This is a self-report measure that may involve recall error. The short time frame used for the travel diary can mean that we might have missed cycling trips of those who do not cycle regularly.

## 5. Conclusions

We have identified the distribution of home-based cycling distances and their variation according to trip purpose and socio-demographic attributes using large-scale travel survey data collected in Australia. We found that cycling distances differed considerably by trip purpose (utilitarian, commuting and recreation). An important implication of the findings is that purpose-specific buffer sizes need to be employed in order to capture environmental attributes related to cycling. Another novel contribution of the study is that it presented a range of distances that may be adopted as a buffer size for future studies on environmental correlates of cycling. The distances obtained in this study may be also used in assessing bikeability, a composite measure indicating to what extent a certain area is cycle friendly. Future research can develop purpose-specific bikeability indices building on the distances obtained in this study.

## Figures and Tables

**Table 1:** Classification of cycling trips by purpose.

Trip Purpose	Purposes Reported in the Travel Diary
Utilitarian	Eat/drink, accompany someone, buy something, get on/off public transport, medical/dental purposes, religious activity, socialising, volunteering or community activity, engage in a personal business, pick-up or deliver something, pick-up or drop-off someone, visit someone
Commuting	Education, work-related
Recreation	Recreation (exercise), leisure activity
Other	Not stated, other purpose, unknown purpose

**Table 2:** Characteristics of study participants who reported home-based cycling on the survey day.

	Number of Cyclists (%)	Number of Survey Participants	Proportion of Cyclists ^1^
Gender			
Men	1115 (67%)	35,194	3.2%
Women	561 (33%)	37,948	1.5%
Age group			
Younger (20–39)	672 (40%)	25,589	2.6%
Middle-aged (40–59)	691 (41%)	30,954	2.2%
Older (60–74)	313 (19%)	16,599	1.9%
Employment status			
Working	1342 (80%)	55,358	2.4%
Not working	334 (20%)	17,784	1.9%
Geographic region			
Metropolitan	1463 (87%)	61,763	2.4%
Regional	213 (13%)	11,379	1.9%
State			
Victoria	1110 (66%)	43,364	2.6%
Queensland	566 (34%)	29,778	1.9%
Total	1676 (100%)	73,142	2.3%

^1^ The proportion of cyclists for each category was calculated by dividing the number of home-based cyclists (reported in the left column) by the number of participants who reported at least one trip (reported in the middle column) in the corresponding category.

**Table 3:** Number of home-based cycling trips and their distances by trip purpose, socio-demographic attributes and regions of residence.

	N (%)	Distance of Travel (km)		
		Mean (SD)	Median (P_20_, P_80_)	*p*-Value
Trip purpose				<0.001
Utilitarian	1124 (33%)	3.1 (3.9)	1.8 (1.0, 4.0)	
Commute	1221 (35%)	7.0 (5.8)	5.3 (2.8, 10.2)	
Recreation	1082 (31%)	5.8 (6.8)	3.7 (1.4, 9.0)	
Other	19 (1%)	5.9 (4.5)	5.1 (2.1, 9.6)	
Gender				<0.001
Men	2300 (67%)	6.0 (6.3)	3.9 (1.5, 9.2)	
Women	1146 (33%)	4.1 (4.7)	2.6 (1.2, 6.2)	
Age group				<0.001
Younger (20–39)	1386 (40%)	4.9 (5.1)	3.4 (1.4, 7.6)	
Middle aged (40–59)	1434 (42%)	5.8 (6.2)	3.8 (1.4, 9.0)	
Older (60–74)	626 (18%)	5.2 (6.6)	3.0 (1.1, 7.4)	
Employment status				<0.001
Working	2771 (80%)	5.6 (5.9)	3.7 (1.4, 8.7)	
Not working	675 (20%)	4.3 (5.6)	2.6 (1.1, 5.7)	
Geographic region				0.007
Metropolitan	2987 (87%)	5.4 (5.6)	3.5 (1.4, 8.4)	
Regional	459 (13%)	5.2 (7.1)	2.9 (1.2, 7.0)	
State				<0.001
Victoria	2283 (66%)	5.6 (5.8)	3.7 (1.4, 8.7)	
Queensland	1163 (34%)	4.9 (5.8)	3.0 (1.2, 7.2)	
Total	3446 (100%)	5.3 (5.9)	3.5 (1.3, 8.2)	—

Note: Nonparametric tests (Wilcoxon signed-rank test when there are two groups within the category and Kruskal–Wallis test when there are three groups within the category) were used to assess the statistical significance of differences in the median cycling distances at different levels of each explanatory variable.

**Table 4:** Distances (km) of home-based cycling trips by trip purpose and gender.

Trip Purpose	Gender	N (%)	Mean (SD)	Median (P20, P80)	*p*-Value
Utilitarian	Men	686 (61%)	3.4 (4.5)	2.0 (1.0, 4.3)	0.01
	Women	438 (39%)	2.5 (2.7)	1.6 (1.0, 3.3)	
Commute	Men	852 (70%)	7.6 (6.2)	5.9 (3.1, 11.4)	<0.001
	Women	369 (30%)	5.6 (4.5)	4.5 (2.3, 8.2)	
Recreation	Men	745 (69%)	6.5 (7.0)	4.1 (1.6, 9.7)	<0.001
	Women	337 (31%)	4.4 (6.0)	2.6 (1.0, 6.3)	

Note: *p*-values were obtained from the Wilcoxon signed-rank test.

## Data Availability

The original data presented in the study are openly available at https://discover.data.vic.gov.au/dataset/victorian-integrated-survey-of-travel-and-activity-vista (accessed on 26 November 2024) and https://www.data.qld.gov.au/ (accessed on 26 November 2024).

## Supplementary Materials

The following supporting information can be downloaded at https://www.mdpi.com/article/10.3390/ijerph21121648/s1, Figure S1: Distribution of cycling distances by trip purpose; Table S1: Survey details by region; Table S2: Additional upper thresholds for cycling distances; Supplementary Materials: Random Forest modelling technique.
