# Supplementary material for: Variations in Cycling Distances by Trip Purpose and Socio-Demographic Attributes: Implications for Spatial Scales to Assess Environmental Correlates of Cycling
Source: Int J Environ Res Public Health. 2024 Dec 10;21(12):1648. doi: 10.3390/ijerph21121648 (PMC11675929; doi:10.3390/ijerph21121648)
Supplement: Supplementary file 1 [file ijerph-21-01648-s001.zip › ijerph-3299234-supplementary.pdf]

Table S1: Survey details by region.

<table>
<tr><th>Region</th><th>Survey name</th><th>Survey year/s</th><th>Geographic size of the region (km²)</th><th>Population size (1,000 persons) [a]</th><th>Response rate %</th><th>Total participants</th><th>Eligible participants [b]</th></tr>
<tr><td>Victoria [c]</td><td>VISTA [d]</td><td>2012 - 2020</td><td>7,713</td><td>4,522</td><td>50</td><td>78,978</td><td>1,110</td></tr>
<tr><td>South East Queensland [e]</td><td rowspan="4">QHTS [f]</td><td>2017 - 2020</td><td>8,101</td><td>2,939</td><td rowspan="2">Not reported anywhere</td><td>36,264</td><td>400</td></tr>
<tr><td>Toowoomba</td><td>2019</td><td>498</td><td>126</td><td>3,963</td><td>24</td></tr>
<tr><td>Cairns</td><td>2014</td><td>254</td><td>139</td><td>51</td><td>5,888</td><td>102</td></tr>
<tr><td>Rockhampton [g]</td><td>2014</td><td>659</td><td>93</td><td>55</td><td>5,420</td><td>40</td></tr>
</table>

[a] Based on Australian Bureau of Census (ABS) data on 2011 population.
[b] Adult aged 20-74 and reported at least one home-based cycling trip on the survey day.
[c] Melbourne metropolitan and surrounding regional cities (Geelong, Ballarat, Bendigo, Shepparton, and Traralgon).
[d] Victorian Integrated Survey of Travel Activities.
[e] Statistical divisions of Brisbane, Gold Coast, and Sunshine coast.
[f] Queensland Household Travel Survey.
[g] Rockhampton and Yeppoon urban centers.

Table S2: Additional upper thresholds for cycling distances.

	Distance of travel (km)		
	P_{75}	P_{80}	P_{85}
Trip purpose			
Utilitarian	3.3	4.0	5.0
Commute	9.0	10.2	11.7
Recreation	7.4	9.0	10.4
Other	7.0	9.6	12.9
Gender			
Men	7.9	9.2	11.0
Women	5.1	6.2	7.3
Age group			
Younger (20-39)	6.3	7.6	8.8
Middle aged (40-59)	7.7	9.0	10.4
Older (60-74)	6.6	7.4	9.7
Employment status			
Working	7.5	8.7	9.8
Not working	4.9	5.7	7.2
Geographic region			
Metropolitan	7.2	8.4	9.7
Regional	6.2	7.0	8.8
State			
Victoria	7.5	8.7	9.9
Queensland	6.0	7.2	9.0
Total	7.0	8.2	9.7

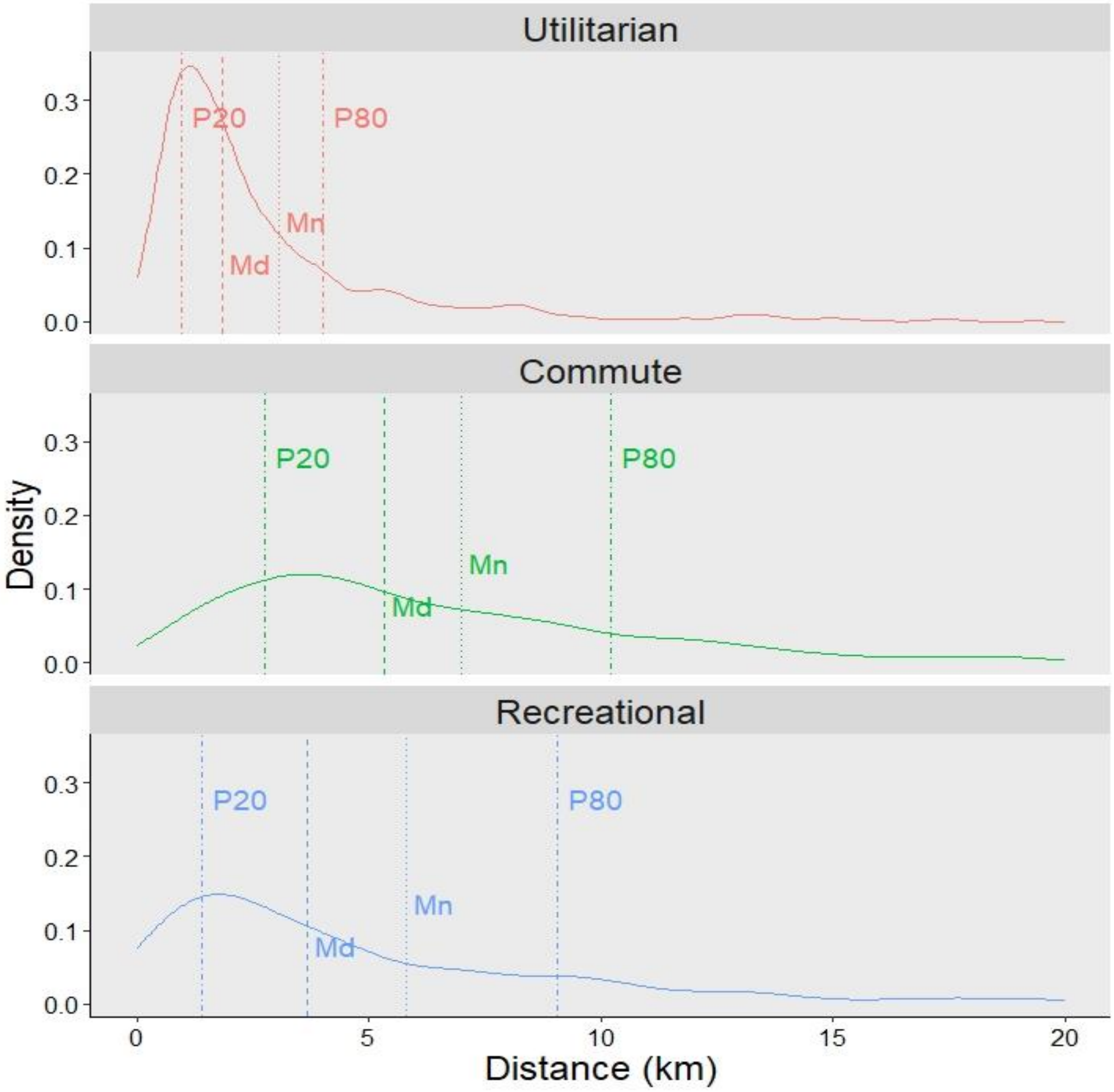

Mn = mean distance, Md = median distance, P_{20} = 20th percentile distance, P_{80} = 80th percentile distance.

Figure S1: Distribution of cycling distances by trip purpose.

Note: Density represents the likelihood or relative frequency of observing a trip of a given distance, with the given purpose.

Supplementary Material: Random Forest modelling technique

This supplementary section provides an overview of the Random Forest modelling technique. The fundamental building block of a Random Forest model is a decision tree. The decision tree modelling involves successively identifying the most important (i.e., most discriminating) explanatory variable and its optimal cut-off value [47]. The focus is on creating two distinct subgroups (denoted by notes in the tree diagram), with the sample being split into two subgroups in a way that can maximize the contrast between them. This process continues for each subgroup. In a decision tree, an explanatory variable's level of importance can be determined by assessing how much variability of the outcome variable is reduced (i.e., homogeneity is improved) due to the partitioning made by that variable.

The Random Forest modelling involves the creation of an ensemble of decision trees, each of which is built on an independent sample of cases chosen from the dataset with replacement (i.e., bootstrapped samples). The Random Forest model combines the outcomes of multiple decision trees to produce a single result. At each split of a tree in the Random Forest model, only a random subset of explanatory variables is considered as split candidates. This facilitates each explanatory variable, even the weakly correlated ones with cycling distance, with more chance to influence the outcome variable compared to a single decision tree.

The algorithm used to construct the random forest model for cycling distance variable in the current study is described below.
Input parameters:

- *n = size of a bootstrap sample of cycling trips (by default, equal to the number of observations in the original dataset) = 3,126,*
- *p = number of explanatory variables of cycling distance = 6,*
- *m = number of explanatory variables considered as split candidates for a split in a decision tree (equal to rounded down $\sqrt{p}$ by default) = 2,*
- *b = number of decision trees forming the Random Forest = 500 (default value in 'ranger' package in R).*

Step 1 (Create Bootstrap Datasets): Generate b number of random samples consisting of n trips selected with *replacement***.** These are called *bootstrap datasets***.** On average, each bootstrap dataset is expected to use two-thirds of the trips in the original dataset [40]. The remaining one-third of observations are called *out-of-bag (OOB) observations*.

Step 2 (Build Decision Trees): Construct a decision tree corresponding to each bootstrap dataset to predict trip distance; this will result in b number of decision trees altogether. Use a random subset of m explanatory variables at each split of the tree (This is known as *feature bagging*). The trees are grown as deep as possible without pruning.

Step 3 (Predict OOB Distances): Obtain the OOB predicted distance for each trip in the original dataset. The trees where a trip is OOB are used to predict the distance of the trip. On average, each trip will be OOB in b/3 trees (see Step 1), and each trip will have b/3 predicted distances. The average of these distances is treated as the final predicted distance of trip by the Random Forest.

Step 4 (Estimate the test Error): Estimate the test (OOB) error of the Random Forest model using the following mean square error formula:

$$OOB\ MSE\ = \frac{1}{n}\sum_{i=1}^{n}(Observed\ distance\ of\ i^{th}\ trip\ - Predicted\ distance\ of\ i^{th}\ trip)^2$$

Step 5 (Estimate variable importance): Residual sum of squares (RSS) (see the equation below) can be used to calculate variable importance. RSS is calculated for all nodes in all the trees and the average reduction in RSS due to splits over a given explanatory variable is used to estimate its importance. Nembrini, et al. [48] present improved approach to estimate the variable importance (i.e., actual impurity reduction).

$$RSS\ = \sum_{i=1}^{n}(Observed\ distance\ of\ i^{th}\ trip\ - Predicted\ distance\ of\ i^{th}\ trip)^2$$

Additional details of this algorithm can be found in standard statistical learning textbooks and articles listed below:

- Breiman, Friedman, Olshen and Stone [47]
- James, Witten, Hastie and Tibshirani [40]
- Liang, et al. [44]
- Nembrini, König and Wright [48]
